# Clonal mutations in primary human glial tumors: evidence in support of the mutator hypothesis

**DOI:** 10.1186/1471-2407-7-190

**Published:** 2007-10-09

**Authors:** Anjan Misra, Parthaprasad Chattopadhyay, Kunzang Chosdol, Chitra Sarkar, Ashok K Mahapatra, Subrata Sinha

**Affiliations:** 1 Dept. of Biochemistry, All India Institute of Medical Sciences, New Delhi, India; 2 Pathology, All India Institute of Medical Sciences, New Delhi, India; 3 Neurosurgery, All India Institute of Medical Sciences, New Delhi, India; 4 Barrow Neurological Institute, St. Joseph's Hospital & Medical Center, Phoenix, AZ, USA

## Abstract

**Background:**

A verifiable consequence of the mutator hypothesis is that even low grade neoplasms would accumulate a large number of mutations that do not influence the tumor phenotype (clonal mutations). In this study, we have attempted to quantify the number of clonal mutations in primary human gliomas of astrocytic cell origin. These alterations were identified in tumor tissue, microscopically confirmed to have over 70% neoplastic cells.

**Methods:**

Random Amplified Polymorphic DNA (RAPD) analysis was performed using a set of fifteen 10-mer primers of arbitrary but definite sequences in 17 WHO grade II astrocytomas (low grade diffuse astrocytoma or DA) and 16 WHO grade IV astrocytomas (Glioblastoma Multiforme or GBM). The RAPD profile of the tumor tissue was compared with that of the leucocyte DNA of the same patient and alteration(s) scored. A quantitative estimate of the overall genomic changes in these tumors was obtained by 2 different modes of calculation.

**Results:**

The overall change in the tumors was estimated to be 4.24% in DA and 2.29% in GBM by one method and 11.96% and 6.03% in DA and GBM respectively by the other. The difference between high and lower grade tumors was statistically significant by both methods.

**Conclusion:**

This study demonstrates the presence of extensive clonal mutations in gliomas, more in lower grade. This is consistent with our earlier work demonstrating that technique like RAPD analysis, unbiased for locus, is able to demonstrate more intra-tumor genetic heterogeneity in lower grade gliomas compared to higher grade. The results support the mutator hypothesis proposed by Loeb.

## Background

In normal germ-line and somatic cells, DNA synthesis and cell division are tightly controlled by various genes, which help in the maintenance of genetic stability. Defect in any of these genes results in accumulation of mutations in the cells. Since an overwhelmingly large portion of the genome is non-coding, cells might tolerate large number of mutations in these regions of the genome. If mutations occur at a critical point in the coding region of gene(s) involved in the maintenance of cellular control on proliferation, DNA repair and differentiation, it might lead to development of a cancerous cell.

Loeb [1] proposed the mutator hypothesis, which stated that cancer cells would accumulate a large number of mutations without influencing the phenotype of a cell. These mutations would arise early in tumor development. In contrast, the persistence of the mutator phenotype would be unnecessary, if not detrimental, to the clonal overgrowth during the final stages of tumor progression [2]. The mutator hypothesis was based on the observations of numerous mutations in different types of tumors, which could not be explained by the mutation rates of normal somatic cells. We felt that determination of the extent of clonal mutations in different stages of neoplasia would provide experimental evidence towards verifying the mutator hypothesis.

A comparison of the DNA fingerprinting pattern of tumors with constitutional DNA from the same patient would identify alterations in tumor genomes in a manner unbiased for locus. Previous reports [3-5] have demonstrated the utility of multi-locus VNTR probes for this purpose. Our group [6,7] and others [8] have demonstrated 'clonal' genomic changes in the genomes of brain and lung tumors by RAPD analysis. By 'clonal' changes we refer to those mutations that do not influence the tumor phenotype. We have identified alterations in DNA by RAPD analysis, a DNA fingerprinting method that does not select for specific genomic locus; therefore, we considered these changes as 'clonal' mutations. Since most of the human genome is non-coding and our selection of RAPD primers was arbitrary, the majority of the bands amplified by RAPD analysis represent the non-coding regions of the genome. Such alterations would differ from the changes identified using locus specific probes or other methods, typically used to look for alterations in known oncogenes or tumor suppressor genes. We have shown that RAPD analysis can demonstrate extensive intra tumor genetic heterogeneity [7,9]. We had also observed that the extent of heterogeneity demonstrated by primers that did not select for loci capable of critically influencing tumor phenotype, was more in DA than the GBM [7].

Glial tumors are the commonest primary brain tumors and second highest cause of mortality by cancer in young adults (after hematological malignancies). According to the WHO classification glial tumors of astrocytic origin are of four grades, ranging from the least aggressive grade I (pilocytic astrocytomas) to the grade II (low grade diffuse astrocytoma (DA)), the grade III (anaplastic astrocytoma (AA)) and the most aggressive grade IV (GBM). Low grade tumors can recur as higher grades [10].

Several alterations in specific oncogenes and tumor suppressor genes in glial tumors have already been determined. These include, amongst others, p53 mutations, EGF receptor gene amplification, changes in the p21 gene, and consistent alterations in loci on chromosome 10 and in the 17p13.3 locus [11-13]. These changes as well as chromosomal alterations and aneuploidy are more frequent in GBM. But the overall extent of clonal changes in glioma genome has not been reported. Recently, an inverse correlation between genetic aberrations and malignancy grade was reported in ependymal tumors [14]. Moreover, colorectal cancers with a high frequency of point mutations displayed a comparatively stable karyotype [15]. Whether similar is true for tumors of astrocytic origin is not known.

In this study we tried to determine the extent of mutations in low and high grade primary human tumors of astrocytic origin. Any attempt to quantify the overall changes in a tumor genome would have its limitations. However, we have tried to clearly define the assumptions and delineated the limitations as precisely as possible.

## Methods

### Tumor sample preparation and DNA isolation

This study was approved by the institutional ethics committee. Tumor and blood samples were obtained from patients with prior informed consent from each patient. Surgically resected tumor samples were collected from the neurosurgery operation theatre of All India Institute of Medical Sciences, India. All cases were primary tumors and had received no prior chemo or radio therapy. No recurrent tumors were taken. Tissue was snap frozen immediately after surgery and frozen sections cut from the entire tumor. Every 15^th ^section was stained with toluidine blue and examined microscopically. Regions of the tumor having more than 70% neoplastic cells (rest necrosed tissue or vasculature, inseparable from the tumor cells) were scraped and used for preparation of DNA. Large tumors were compartmentalized to segments of 50 sections each [7] and DNA from only one such segment (selected at random) was used in the study. Leucocytes of the same patients were the source of corresponding constitutional DNA. DNA from both tumor and blood were extracted by Proteinase K digestion and Phenol:Chloroform extraction [16].

### RAPD analysis

10-mer primers of random but definite sequences of 50–80% GC content were purchased from Genosys, Texas, USA; and used with varying magnesium chloride concentrations and annealing temperature (Table 1) for PCR amplification. In RAPD a single primer serves as both the forward and reverse primer [17,18]. A set of 15 such primers was used in this study. A band was amplified when complementary sequences capable of primer binding were present in genome on opposite DNA strands within a PCR amplifiable distance. For each 20 μl PCR reaction, 100 ng genomic DNA, 50 pM of primer, 0.75 U of Taq DNA polymerase, 200 μM of dNTP (both from Genie, Bangalore, India) each and specified concentration of magnesium chloride were used. After Hot-start, PCR was done for 33 cycles of 1 min. at 94°C, 1 min. at specified annealing temperature and 2 min. at 72°C with 1 sec extension in each cycle in either a MJ Research mini thermalcycler (Model No. PTC 150) or Perkin Elmer DNA Thermocycler (Model No. 480). PCR conditions were set to obtain a reproducible and distinct banding pattern. The annealing temperature varied from 22°C to 48°C and MgCl_2 _concentration varied from 1.5 mM to 4.5 mM. PCR products were subjected to 1.5% agarose gel electrophoresis in 1× TAE buffer, visualized by ethidium bromide staining and documented. The model of the thermal cycler was not changed for a particular primer during the course of the study. Each RAPD reaction showing altered bands in tumor was confirmed by at least two independent experiments.

**Table 1:** RAPD primer sequences and PCR conditions

**Primer ID**	**Primer Sequence**	**Magnesium Chloride Concentration (mM)**	**Annealing Temperature (°C)**
50/09	5' AGAAGCGATG 3'	1.5	28
50/24	5' GTTAGTGGCA 3'	3.0	22
60/39	5' CGCTGTTACC 3'	3.0	28
70/09	5' TGCAGCACCG 3'	3.0	42
70/31	5' GCCCCTCTT G 3'	3.0	36
70/34	5' GGACCGCTAG 3'	3.0	33
70/40	5' CGCAGACCTC 3'	4.5	33
80/03	5' CCATGGCGCC 3'	3.0	48
80/06	5' GCACGGAGGG 3'	4.5	42
80/32	5'GCCCCATGCG 3'	4.5	48
80/35	5' CACCTGCCGC 3'	1.5	45
80/37	5' CGCCAGGAGC 3'	4.5	42
RE-05	5' GCGAATTCCG 3'	3.0	37
RE-06	5' CGGAATTCCG 3'	3.0	37
RE-21	5' GGCTGCAGCG 3'	3.0	45

### Scoring

Scoring was done by comparing the RAPD profile of every tumor with each primer to its paired constitutional DNA RAPD profile of the same primer [7]. In order to rule out artifacts caused by efficiency of amplification or sample loading, only those bands were scored for alterations where the preceding and succeeding bands had comparable intensities in the tumor and paired normal DNA. A new band in tumor profile was scored as 'gain', and a band present in normal DNA but not in tumor DNA, was scored as 'loss'. Total number of amplified and altered bands (gain/loss) were counted. To ensure uniformity in scoring, bands showing increased intensity in tumors have not been scored even though that may have resulted in alterations being under reported. RAPD bands in tumors with intensity significantly lower (≤ 30%) than the control were scored as loss of band (as some contamination with normal tissue could not be ruled out). This was similar to scoring for loss of heterozygosity reported in literature. Scoring was done by three observers independently. Any difference was resolved mutually.

The size of different bands in the RAPD analysis was calculated with the help of FotoEclipse System (Fotodyne Inc, Wisconsin, USA), Collage version 4 Software. The total length of all the bands amplified as well as the total length of the altered bands in each RAPD reaction was calculated. Mean percentage alteration of DNA for each tumor by each primer and in the groups of DA and GBM were calculated.

### Leucocyte DNA as constitutional DNA

Since it was not possible to obtain corresponding normal brain tissue as a control for tumor DNA, we used blood DNA of same patient as normal DNA. With the assumption that DNA from any normal tissue will essentially be the same and would give same profile, we compared the RAPD profile of leucocyte DNA with buccal mucosal DNA of same person in 6 volunteers. We also collected leucocytes from individuals at intervals of 4–6 months, up to a period of 2 years and determined the reproducibility of the RAPD profile in same individual's DNA over time.

### Precautions for reproducibility

One of the concerns regarding data obtained by RAPD analysis regards reproducibility of data. In this study extensive precautions were taken to maintain consistency in experimental results as discussed in our earlier reports [7,9]. Essentially, only those primers that demonstrated clear and reproducible RAPD amplification patterns were used. We took precautions to maintain DNA and primer quality and also observed the precautions for scoring that are described earlier in the paper.

### Southern Blot analysis for determining the uniqueness of the altered band

Five randomly selected altered bands were hybridized to the RAPD profile of the tumor and control DNA from which they were derived. This was done to determine whether an altered band was unique in its corresponding RAPD profile or whether the alterations were caused by minor changes in the microsatellite repeat sequences of any of the other bands in the same sample. After the RAPD profile of the tumor and corresponding normal DNA was resolved by agarose gel electrophoresis, it was transferred to a nylon membrane (Hybond N, Amersham, UK) by capillary blotting and UV cross-linked to the membrane. The altered band, which had been previously eluted from a gel and purified, was labeled by PCR reaction using P^32^dCTP (NEN, USA). The labeled probe was extracted and purified. Hybridizations were carried out in 5× SSC, 0.5% SDS and 5% dextran sulfate at 62°C over night. The membranes were washed twice with 2× SSC at room temperature, followed by 2 washes in 0.5× SSC at 62°C for 30 minutes each and 2 washes with 0.1× SSC for 2–3 min at room temperature and auto-radiographed by using X-omat XK-5 film (from Kodak).

### Cloning and sequencing of the altered bands

Five altered bands identified by RAPD analyses were further analyzed by cloning, sequencing and homology search.

### Estimation of overall extent of alterations in tumor genome

The calculations for determining the extent of genomic changes in tumors were done by two modalities using criteria, which were mutually exclusive. For both these modalities we have made certain assumptions. These assumptions were not complete and carry obvious limitations as stated. However, we believe that these limitations do not challenge the essential validity of our observation.

#### Method I

The basic assumption was that the RAPD primer binding sites were representative of the entire genome in an unbiased manner. Obviously this cannot be true for coding or critical regulatory sequences but are likely to be representative of the non-coding regions of the genome, which any way, form the bulk of the genome. For amplification of a band in RAPD analysis same primer has to bind to complementary sites on both strands. These sites should be at a distance suitable for PCR amplification. Because of the low stringency employed in the technique, RAPD priming can occur even with 6–8/10 matches in the primer if the 3' end is matched [19]. Hence, a total of 2 such (6–8 base) matches per haploid genome or 4 matches per diploid genome is assumed if a band was observed. Another important assumption was that each band in a RAPD profile actually represents two bands, each amplified from a haploid genome. We felt this as a justifiable approximation because: a) PCR products were resolved by 1.5% agarose gels that cannot separate the 2 alleles (that may vary in size by a few base pairs) as seen in LOH studies. b) Keeping in mind the frequency of single nucleotide polymorphisms (SNPs) and other differences between two haploid genomes (~1 in 1000 bases), two stretches of DNA at same locus have a much greater likelihood of being similar than different. Hence, the minimum information that can be derived from one band in a RAPD profile is that of 12–16 bases (6–8 × 2) on primer binding sites. Any change in the RAPD pattern arose because of loss or creation of a site suitable for RAPD priming. Since annealing of a RAPD primer occurs at a low stringency, and up to 2–4 mismatches can be tolerated under such conditions, it is likely that a band would not be amplified if there were a mismatch at the 3' end of any one of the two primer binding sites. This would be particularly true for an enzyme like Taq polymerase that does not have proof reading activity. If a band was visible in RAPD pattern of normal (leucocyte) DNA but not the tumor (referred as ' loss of band'), it meant mutation at the 3' end of at least one primer binding site on both the alleles (that is alteration at 2 sites). The minimum change required for every new band in a tumor DNA, compared to its corresponding leucocyte DNA (referred to as 'gain' in this paper) would be one mutation, which generates a perfect match, creating a primer binding site. This needs to occur on only one allele. Since the probability of having up to 2–4 point mutations in a 10 mer stretch is low, we have not considered these situations for our calculations. Our calculations are based on only the need for a perfect match at the 3' end of the primer for PCR to occur, assuming that a homologous site already exists within an amplifiable distance from the other primer binding site where the primer can bind in the reverse direction.

A limitation of this approach is that it does not take into account rearrangements that place primer sites in apposition or distance them beyond the limit of RAPD amplification. Also gross chromosomal alterations not affecting closely located primer binding sites will not be reflected. Similarly, amplifications resulting in increased intensity of a band are not reflected in these calculations. However, the extent of the error would be similar in high and low grade tumors resulting a conservative estimate of changes. Hence, while conscious of these limitations, we have calculated the extent of mutations in the following way:

a) Number of amplified bands × 4 = the number of relevant 3' ends (as the same primer acts both as forward and reverse primer and there are two alleles). These are the total number of bases under consideration.

b) Number of lost bands × 2 = mutations complementary to the 3' ends of the primer (this is because while a single mutation altering the base complementary to the 3' ends of a primer will prevent PCR amplification from one haploid genome, a homozygous change with mutations occurring in both the alleles is needed for the absence of a band).

c) Appearance of a new band corresponds to, at least, a single change which creates a perfect match with the 3' end of the primer within an amplifiable distance of a preexisting match i.e. one additional band in the tumor = 1 mutation. Again, it was assumed that a homologous site for primer binding already exists and the single mutation generates only one binding site complementary to the 3' end of the primer.

The values for every leucocyte/tumor pair for each primer were calculated separately.

#### Method II

In the previous method we had only considered mutation at 3' end of primer binding site as a relevant reason for observing loss/creation of a new band. No role of genomic rearrangements, large losses etc. were considered. In this method, we calculated the extent of change taking the other extreme assumption that all changes are due to rearrangements or losses and none were due to mutations at the bases annealing to the 3'end of the primer. The size of each RAPD amplified bands (33 sample pairs, each with 15 different primers) was determined (as mentioned above). The length of DNA amplified by each primer from each patient's leucocyte DNA was individually determined and added up to estimate the total length of DNA amplified by all the primers for each tumor. The length of each altered fragment (lost/gained) was determined and added up to estimate the total length of the DNA fragments altered in a tumor. Mean percentage alteration in each tumor by each primer and in DA and GBM were calculated.

A limitation by this method is that loss/gain of a larger band on the RAPD profile may not necessarily represent a larger genomic change than in case of a smaller band. However, this limitation would be true for both high and low grade tumors.

The basic difference between both the methods is that while the first method considers only those bases, which bind the primer, the second method considers only the region, which gets amplified by the PCR reaction. It is also assumed (for both the methods) that bands observed on RAPD are essentially distinct from each other. This is based on experiments (discussed below), where in a number of different tumor-primer combinations, an altered band was hybridized to the same RAPD profile, did not hybridize to any other band.

### Statistics

Two tailed t test was used to compare extent of aberrations in DA and GBM by both the methods.

## Results

**Figure 1:**
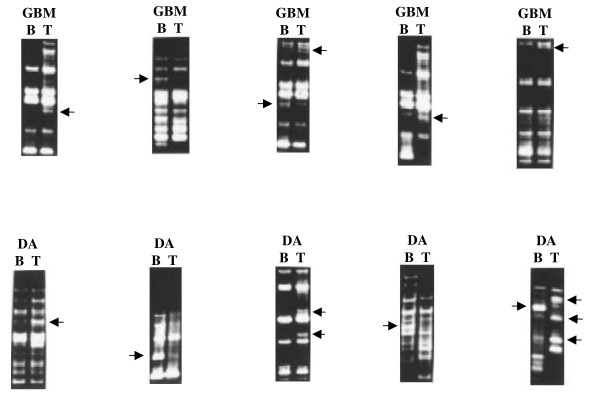
RAPD patterns of different tumors compared to their corresponding normal DNA. Leucocytic DNA RAPD profile is marked as **B **and tumor DNA by **T**. Grade II astrocytoma is indicated as DA and Glioblastoma multiforme is indicated as GBM. Arrow indicates an altered band. From left to right, upper panel: 80/32 with 154; 80/03 with 278; 60/39 with 195; 70/09 with 195; 50/09 with 172; lower panel: RE21 with 173; RE05 with 195; primer 70/09 with tumor 175; RE06 with 154; RE21 with 175.

The PCR amplified bands varied between 3 Kb to 400 bp in size, the majority of bands were less than 1 Kb size (Figure 1). When the RAPD profile of normal and tumor DNA was compared, differences were observed within a number of blood-tumor pairs. This is similar to what we had reported earlier [6,7].

**Figure 2:**
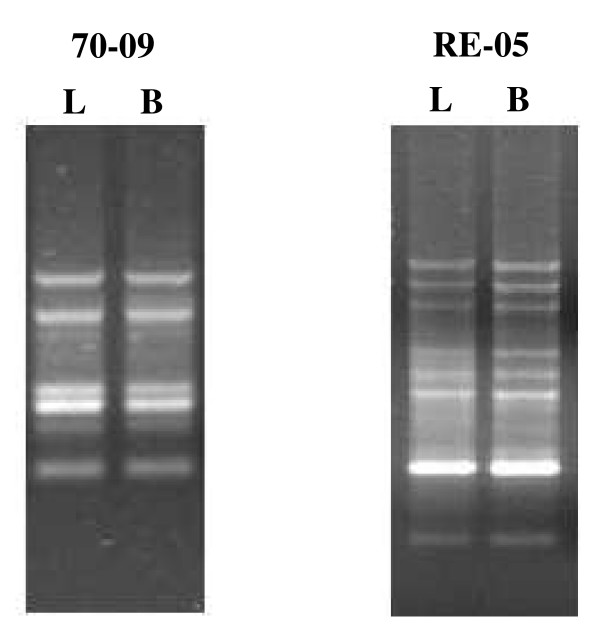
Comparison of the normal RAPD profiles obtained from DNA extracted from buccal scraping and the peripheral blood leucocytes of a healthy volunteer with different primers. The primers number is indicated on the top of the figure. B and L indicate buccal mucosal and leucocyte DNA respectively.

Leucocyte DNA was taken as a representative of the normal DNA. The stability of RAPD profile for each primer was indicated by identical RAPD pattern obtained from DNA extracted from buccal scraping and the peripheral blood leucocyte in healthy volunteers (Figure 2). Also the RAPD profile was stable when leucocyte DNA was obtained from the same individual on intervals of 4–6 months over periods of up to two years (data not shown).

The RAPD results are summarized in Tables 2, 3, 4, 5. Thirty two tumors (out of 33) showed genetic alteration(s) when studied with this panel of 15 RAPD primers. These included loss of band/s, gain of band/s, gross change in intensity of bands or any combination of them. Approximately half of these alterations were loss of a band in the tumor, which is present in paired constitutional DNA of same patient. Tumors of same histological grade showed different degrees of genetic alteration (e.g. tumor No. 137 vs. 194; tumor No. 154 vs. 159); also some primers were more efficient in identifying changes (primer No. 50/09, 80/32) than others (primer No. 70/31, 80/37). In some cases extensive alterations were demonstrated by one primer but not by others (eg. tumor No. 137, primer No. 50/09 and 60/39).

**Table 2:** Alterations in Low grade diffuse astrocytomas (DA) calculated by method I

**Tumor No**.	**Number of altered bands/total bands amplified**	**Net change**	**% change of bands in different tumors**
108	L10G4/204	24/816	2.94
132	L6G5/101	17/404	4.21
137	L16G16/172	48/688	6.98
150	L4G4/100	12/400	3
172	L4G3/71	11/284	3.88
175	L9G18/121	36/484	7.44
194	L2G0/94	4/376	1.06
195	L20G12/140	52/560	9.28
235	L2G5/211	9/844	1.06
244	L0G5/188	5/752	0.66
245	L3G8/191	14/764	1.83
277	L8G11/138	27/552	4.89
278	L8G8/76	24/304	7.89
279	L7G3/139	17/556	3.06
280	L10G7/129	27/516	5.23
284	L4G4/136	12/544	2.21
285	L9G16/133	34/532	6.39

The number of lost/gained bands are indicated by **L**/**G**, while the total number of bands amplified in the corresponding normal DNA is given as the denominator.

**Table 3:** Alterations in Glioblastoma multiforme (GBM) calculated by method I

**Tumor No**.	**Number of altered bands/total bands amplified**	**Net change**	**% change of bands in different tumors**
115	L7G9/137	23/548	4.19
123	L1G2/104	4/416	0.96
125	L4G0/108	8/432	1.85
127	L3G0/141	6/564	1.06
129	L4G5/166	13/664	1.96
131	L0G0/179	0/706	0
154	L10G7/148	27/592	4.56
158	L5G2/181	12/724	1.65
159	L1G0/166	2/664	0.30
173	L3G1/133	7/532	1.31
174	L9G5/115	23/460	5
188	L4G2/141	10/564	1.77
238	L7G8/174	22/696	3.16
241	L7G9/201	23/804	2.86
282	L5G3/91	13/364	3.57
287	L2G9/137	13/548	2.37

The number of lost/gained bands are indicated by **L**/**G**, while the total number of bands amplified in the corresponding normal DNA is given as the denominator.

**Table 4:** Alterations in Low grade diffuse astrocytomas (DA) calculated by method II

**Tumor No**.	**Total Length of Tumor DNA (Kb) studied**	**Total Length of Change in tumor DNA (kb)**	**% alteration of DNA in different tumors**
108	116.4	8	6.9
132	90.3	10	11.1
137	123.3	40.2	32.6
150	100.2	4.3	4.3
172	106.6	16.1	15.1
175	123.3	24	19.5
194	104.4	2.6	2.5
195	123	26.3	21.4
235	201.8	9.1	4.5
244	174.3	3.2	1.9
245	202.1	5.3	2.6
277	139.5	13.8	9.9
278	117.1	23.2	19.9
279	168.6	8.1	4.8
280	175.5	16.8	9.6
284	161.5	9.3	5.7
285	125.1	22.3	17.8

Mean percentage alteration in each tumor by each primer was calculated

**Table 5:** Alterations in Glioblastoma multiforme (GBM)calculated by method II

**Tumor No**.	**Total change in tumor DNA (kb)**	**Total Length of Tumor DNA (Kb) studied**	**% alteration of DNA in different tumors**
115	17.5	119.2	14.3
123	3.6	112.6	3.2
125	4	103.9	3.8
127	3.4	100.8	3.4
129	6.3	134.9	4.7
131	0	145.6	0
154	12.8	126	10.1
158	6.5	167.9	3.8
159	2	138.2	1.4
173	2.2	114.7	2
174	11	97.4	11.3
188	8	131.6	6
238	12.8	174	7.3
241	9.6	185.6	5.2
282	7.3	88.4	8.2
287	3.6	141.2	2.5

Mean percentage alteration in each tumor by each primer was calculated.

**Figure 3:**
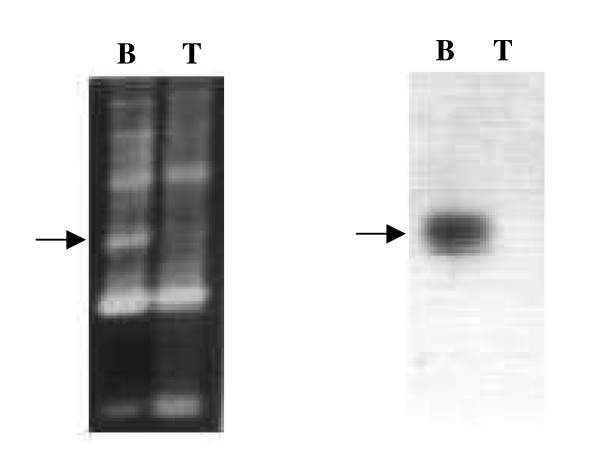
Representative Southern hybridization experiment for detecting homology of the altered band with other bands in the RAPD profile. When RAPD analysis was performed with tumor no 195 and corresponding normal DNA with primer RE 05, loss of a normal band in tumor tissue was detected. Probe made by labeling this band (eluted from the gel in a previous experiment), did not hybridize to any other band in either tumor or normal DNA.

Five altered bands (80/35/127B, RE06/125B, 80/35/195T, RE05/195B and 70/09/173T) chosen randomly were tested for homology to other bands in the RAPD profiles (both tumor and corresponding normal DNA) from which they were amplified. Southern hybridization revealed that these altered bands did not hybridize with any other band in that RAPD profile. This indicated that these altered bands were not part of any other band in the profile or were not result of minor changes in microsatellite repeat sequences in other amplified band. A representative experiment is shown in Figure 3.

These altered bands were further confirmed for their uniqueness by cloning and sequencing. They were cloned in either pGEMT, pGEMT-Easy, pBlueScript or pUC II vectors. Cloned fragments were further analyzed by Southern hybridization with the corresponding RAPD eluted bands (accession numbers AF264057, AF264058, AF264059, AF264060 and AF264061). Unlike the results in AP-PCR [20,21] where the altered bands were resolved by polyacrylamide gel electrophoresis, no microsatellite repeats were observed in any of these cloned fragments. However, the nature of the altered fragments suggested that the sequences preponderantly were of regions likely to be involved in recombination. One fragment had homology with retroposon HERV-K [22] and another to L1 repetitive element and Chi-core sequences. These are sequences present in the normal human genome as identified by BLAST search. These changes are not common among tumors. Nor are they part of coding sequences. Hence they indicate alterations in tumor DNA that are not selected for the locus. The details of the characterized altered fragments are as follows:

**i) Altered fragment (80/35/127B) (accession no. AF264058) **showed homology with parts of retroposons of human endogenous retrovirus origin (accession nos. X07417, X07418 and X07419). Retroposons are repeated sequences and are widely distributed in the human genome. BLAST homology also showed several stretches of various lengths of this altered sequence interspersed throughout the human genome (accession nos. AV373514, Z73358, AB000381, AC000387, U91326, D84394, Z69710, Z69710, AC002992, Z11740, Z82196 and Z83841).

**ii) Altered fragment (RE06/125B) (accession no. AF264061) **showed partial homology to L1 repetitive sequence. L1 sequences are highly repetitive interspersed sequence, an estimated 100,000 L1 elements are present in the entire human genome. These sequences comprise as much as 17% of the human genome by weight (Smit AFA, 1996, Curr.Opin. Genet. Devel., 6, 743–48).

**iii) Altered fragment (80/35/195T) (accession no. AF264060) **On homology search, the complete stretch has no homology with sequences in the public domain database. However, it has 6 nucleotides of the Chi core octanucleotide (GCTGGTGG) sequence of E.coli. Chi-core octanucleotide sequence is present at the DNA break points in human tumors. Chi core influences the probability of the nearby rearrangement or it alters the regulation of oncogene transcripts once rearrangement occurs (Krowczynska et al., 1990, Nucleic Acids Res., 18, 1821). Many such Chi homology sequences repeated in the human genome have been used for DNA fingerprinting (Ehtesham et al., 1990, Ind. J. Biochem. and Biophysics. 27; 275–279).

**iv) Altered fragment (RE05/195B) (accession no. AF264059) **shows no homology in the human genome sequence in the public domain data base. However it is an AT rich sequence and AT rich sequences are reported to be prone to rearrangement by recombination.

**v) Altered fragment (70/09/173T) (accession no. AF264057) **shows no homology in the human genome sequences in the public domain till date. This sequence also showed an AT rich sequence.

### Calculations of Genomic Change based on Method I

The changes observed in Method I are listed in tables 2 (for DA) and 3 (for GBM). For every tumor-primer combination, the number of lost/gained bands are indicated by **L**/**G**, while the total number of bands amplified in the corresponding normal DNA is given as the denominator.

The number of alterations (3' ends mutated) = Number of lost bands × 2 + number of gained bands × 1.

The total number of 3' ends studied is = total number of bands amplified in normal DNA × 4.

The extent of alterations varied from 0.66% to 9.28% in DA, with a mean value of 4.24 ± 2.62 (standard deviation or SD). In GBM it varied from 0% to 5%, the mean value 2.29 ± 1.49 (SD). The alterations were more in DA and statistically significant (two tailed p = 0.014).

### Calculations of Genomic Change based on Method II

The results are summarized in table 4 (for DA) and 5 (for GBM).

The ranges of alterations in DA and GBM were 2.3% to 27.8% and 0% to 20.2% respectively. Only one tumor (No. 131) showed no genetic change. The mean percentage alteration in DA was 11.96 ± 7.44 (SD) and in GBM was 6.03 ± 5.24 (SD) (Tables 4 and 5). Changes in DA was significantly higher than in GBM (two tailed p = 0.013).

## Discussion

There is considerable debate over the role of genomic instability as a cause or consequence of tumorigenesis. By the time a tumor is clinically manifest, it has survived a checkered history of multiple rounds of cell division, cell death, clonal expansion and selective growth of some surviving clones. The altered nature of the tumor cells, and indirectly, the natural history of the tumor would be reflected in the magnitude of the overall genetic changes in a tumor. In this work RAPD-PCR of tumors and its comparison with the normal DNA has been used as a measure of genomic instability.

Genomic instability in the form of chromosome instability, aneuploidy, LOH, microsatellite repeat alterations are seen in almost all types of neoplastic and preneoplastic cells. All these mutations have different implications in the tumor development and progression. However the cause of genomic instability is still debated. Some authors find aneuploidy as the primary cause of the genomic instability in the tumors [23,24]. Ninomiya et al [25] have found chromosomal instability and LOH to play role in the development of lung cancer. Goel et al [26] have characterized the role of chromosomal and microsatellite instability in the progression of colon cancer. In other systems, if one restricts to microsatellite repeat alterations, there has been some evidence linking genomic instability to tumor progression [27,28]. Microsatellite instability was found to be associated with aggressive colorectal carcinoma [29] and ovarian carcinoma [30]. However in a broader sense, the association of generalized genomic instability with tumor progression would be somewhat in contradiction with the mutator hypothesis, in which the mutations are phenotypically neutral with no direct role in the progression of the tumors. A mutator phenotype might be a liability to an aggressive rapidly dividing clone arising during tumor progression as extensive mutations might adversely affect the growth properties of these cells [2]. This apparent contradiction can be resolved if it is kept in mind that the assays for genomic instability measure different phenomena, with varying biological implications. For example, in colorectal tumors increased frequency of point mutations is associated with a stable karyotype [15], which is less likely to be associated with progression.

There have been several attempts to estimate the extent of alterations in tumor genomes. Ionov et al [20] used radioactive Arbitrarily Primed PCR (AP-PCR) and PAGE to study genomic mutations in colorectal carcinoma, documenting and characterizing bands altered in tumors. AP-PCR is similar to RAPD except the primers used are longer. They resolved PCR product on PAGE with a higher resolution for band sizes up to 1 kb and observed that altered bands in tumors were identical to the closest normal band except for deletions in repeats like poly A, dinucleotides like CT and CA or trinucleotide repeats. Their estimation of changes in the genome was about 1.3–1.5 per 10^5 ^bases in cancer tissue. This is very different from what we see. In our study, most of the altered bands do not have any homology with any other bands in the same RAPD profile as confirmed by Southern hybridization of the altered bands to the RAPD profile (Figure 3).

Jackson and Loeb [31] estimated ~100,000 genome wide alterations in cancers in the form of base substitutions, deletions, chromosomal translocations, and gene amplifications, and these mutations are found to accumulate as tumors progress. They proposed that the genomic instability caused by these alterations result from a mutator phenotype, which occurs early during tumor development and predisposes the tumor cell to the accumulation of further mutations. They [32] emphasized the importance of considering different types of alterations in genome of malignant cells, like aneuploidy, translocations and amplifications along with those detected by molecular methods like analysis of loss of heterozygosity, microsatellite instability etc. Similarly Kerangueven et al [33] have found extensive genetic diversity from a genome wide scan for loss of heterozygosity. Accumulation of simple random point mutations can lead to carcinogenesis [34]. To predict the relative contribution of mutator mutants in cancer, Beckman and Loeb [35] have developed a mathematical model that quantitatively determines the efficiency of carcinogenesis with and without mutator mutations. Recently Beilas et al [36] have measured random mutations in normal and neoplastic human tissues and have found an elevation in random mutations of at least two orders of magnitude in neoplastic tissues.

There are studies using inter-simple sequence repeat PCR (ISSR-PCR), a DNA fingerprinting method to identify genomic instability in the tumors. Basik et al [37] found that the extent of alterations detected in colorectal cancer using ISSR-PCR had no relationship with the tumor stage at diagnosis and microsatellite instability, though it was associated with loss of heterozygosity. The number of genomic alterations in colorectal cancer cells was more than expected and mean number of alterations per cell was ~11,000 [38]. The lack of association of this phenomenon with tumor grade and observation of similar number of events in colonic polyps and colon carcinoma supports the theory that genomic instability occurring early during malignant change. Stoler et al [39] using ISSR-PCR found genomic instability in invasive breast carcinoma. The mean instability index was 3.08%, which was similar to the mean value observed in colorectal and thyroid carcinoma studied by the same group. Using ISSR-PCR Rai et al [40] showed genome wide instability with the mean instability index of 12% in chewing-tobacco associated oral cancer. However, ISSR-PCR measures only a specific type of regions in genome, between two defined repeats located end to end within a certain distance from each other. The RAPD analysis used in our study targets genomic loci of a different nature and we estimate that the number of overall alterations in tumor genome seems to be greater than earlier appreciated. While the estimated number of alterations in our study is different from that observed using ISSR-PCR, the observation of absence of increased genomic alteration by Basik et al [37] in higher grade tumors is similar to our finding. Both ISSR-PCR and RAPD-PCR are techniques for measuring genomic changes that are likely to be phenotypically neutral.

Tomlinson et al [41] proposed that normal mutation rate is sufficient to explain a large number of mutations in tumors if the number of cell divisions during normal development is taken into account. We were not able to study normal brain tissue as control, and used normal leucocyte DNA. We checked the stability of the RAPD analysis by comparing the RAPD profile of buccal mucosal DNA with the leucocyte DNA from the same volunteer. Also the leucocyte DNA from individuals was obtained in intervals of 4–6 months over a period of two years, and RAPD profile of each sample were identical for the same person. The identical RAPD profile from buccal mucosal and leucocyte DNA, along with stability of leucocyte DNA profile over a period of 2 years, during which there would be a lot of cellular turnover, indicates that the alterations described in this study do not represent alterations accompanying cell division that might occur during normal development. These results, along with the universal forensic practice of taking the DNA fingerprinting profile of leucocyte DNA as representative of normal DNA for comparison with DNA of diverse tissue origins, body fluids etc. justifies the use of blood as constitutional DNA in our study.

Most of the published work till date on the extent of mutations in tumors is on colon carcinogenesis. Our study is on primary human glial tumors of astrocytic origin and we have taken DA and GBM as representative of low and high grade tumors of same cell origin. The mean estimate of alterations observed in tumor genomes varied between 4.42% in DA and 2.29% in GBM by method I and 11.96% (DA) and 6.03% (GBM) by method II. The difference between DA and GBM by both the methods was statistically significant. The increased extent of alterations occurring in tumors of a lower grade differs from the results obtained by some studies using AP-PCR [29,30]. Both RAPD and AP-PCR are basically similar techniques, relying on a single primer to identify sites in both directions on double stranded DNA to prime a PCR reaction. However, there are basic differences in the methods employed by us and by the other researchers, which seems to have resulted in an altogether different form of clonal mutations identified in our study. In previous studies using AP-PCR followed by PAGE, the altered bands (as determined by sequencing) were found to differ from the closest normal counterpart only by small runs of simple repeat sequences. In our case the altered bands did not have any similarity with the normal bands on the RAPD profile. Five of the altered bands, characterized in detail, did not hybridize with any other band in the RAPD profile of either tumor or paired normal DNA (Figure 3). Sequencing showed no microsatellite repeats in these altered fragments. These unique sequences were homologous to some recombinogenic sequences like HERV-K [22], L1 repeat sequence and *Chi*-core sequences (manuscript in preparation) and had the potential to mediate alterations in the genome by repeat mediated recombination and other mechanisms. Thus the changes identified by us are not due to microsatellite repeats being deleted from or added to the nearest normal band. The different nature of changes identified by us could be because of use of agarose gel to separate the PCR amplified bands. Agarose gel has a lower resolution range than PAGE, and while they have been extensively used in RAPD analysis, they do not resolve changes of few base pairs caused by microsatellite expansion and contraction. Hence, the changes detected by us were qualitatively different from the earlier studies. The size of bands studied by us is also different, up to 2.5–3 kb as opposed to much smaller bands studied by other groups. We have also used 10 mer primers, which would be expected to prime more promiscuously than longer primers used by others.

Our observation of a higher number of genetic changes in tumors of lower grade could be a consequence of an increased mutation rate in early tumorigenesis due to acquisition of a mutator phenotype. It is assumed that in absence of selection pressure the number of mutations would be more. However, the mutator phenotype would be at a disadvantage to a more aggressive rapidly growing clone [2] as would be expected in a GBM, especially in a primary GBM. The higher number of mutations could also be a reflection of the longer time taken by a low grade tumor to clinically manifest, compared to a higher grade tumor. Mutations could accumulate in non-dividing cells [42], thus making tumors with low proliferation indices e.g., grade II (DA) tumors accumulating more mutations than grade IV (GBM) tumors. Another factor could be the higher rate of apoptosis in low grade tumors, which would lead to cell populations in DA accumulating more clonal mutations, both as a function of time as well of cell divisions, before the tumor is manifested clinically.

We have earlier demonstrated that RAPD analysis reveals increased intra-tumor genetic heterogeneity in DA compared to GBM [7]. When DNA was independently extracted from consecutive segments of 50 sections each, more differences between the segments of the same tumor were detected in DA compared to GBM. This result was also in concordance with the acquisition of a mutator phenotype early in tumorigenesis, leading to the co-existence of progeny cells with different DNA fingerprinting patterns, especially in low-grade tumors. It would be expected that heterogeneity might give the impression of increased change in a large tumor. In order to exclude such a possibility, we used DNA made from only one segment of 50 consecutive sections in each tumor used in our study. Similarly another study by our group [43] has observed very significantly higher level of loss of heterozygosity (LOH) in the hMLH1 gene locus in DA compared to GBM using microsatellite markers, correlating with our earlier result which showed more intra tumor genetic heterogeneity in low grade gliomas.

The uniqueness of our study lies in the high estimated number of mutations. Also the estimated number of clonal mutations is greater than earlier reported, and not just restricted to microsatellite repeat sequences length. The extent of such changes is more in the low grade tumor type (DA) than in high grade GBM. Our evidence is in favor of dissociation of the dynamics of clonal mutations that are phenotypically neutral from those genetic changes leading to a phenotype with growth advantage. This is in concordance with the applicability of the mutator hypothesis. A further study comparing primary and secondary GBM would provide more evidence for its applicability in glial tumors. Similar approaches can also be used to study the role of mutations that do not have phenotypic consequences in other tumor types as well.

## Conclusion

In this study, using locus non selective RAPD fingerprinting method, we studied the genomic instability in the astrocytic tumors of WHO grade II (DA) and grade IV (GBM), using leucocyte DNA of the same patient as control. The alterations detected were non coding sequences, not common among tumors, and indicate that they are not selected for specific locus. The quantitative estimation of the overall genomic changes detected in these tumors was done by two mutually exclusive modes of calculation, yielding similar results. The results demonstrate the presence of extensive clonal mutations in gliomas, more so in the grade II astrocytomas. This is in line with the mutator hypothesis – that, those mutations/genetic alterations that do not have a significant phenotypic correlation are likely to be more frequent in low grade tumors. This is in consistent with our earlier work demonstrating that technique like RAPD fingerprinting analysis is able to demonstrate extensive intra-tumor genetic heterogeneity in all grades of gliomas, more so in lower grade as compared to higher grade. Our results showed the acquisition of a mutator phenotype early in tumorigenesis and support the mutator hypothesis proposed by Loeb.

## Competing interests

The author(s) declare that they have no competing interests.

## Authors' contributions

AM carried out the samples processing, RAPD PCR standardization, study design, RAPD analyses, cloning, sequence alignment and drafted the manuscript. PC participated in sequence alignment and performed the statistical analysis and helped write the manuscript. KC carried out the sample processing, RAPD analysis and helped write the manuscript. CS carried out the pathological analysis of the tumor samples, including determining the proportion of tumor and normal cells and helped write the manuscript. AKM carried out the tumor samples selection and analysis and helped write the manuscript. SS conceived the study, helped in study design, statistical analysis, coordination and helped write the manuscript.

All the authors have read and approved the final version of the manuscript.

## Pre-publication history

The pre-publication history for this paper can be accessed here:


